# TGF-β Enhanced IL-21-Induced Differentiation of Human IL-21-Producing CD4^+^ T Cells via Smad3

**DOI:** 10.1371/journal.pone.0064612

**Published:** 2013-05-31

**Authors:** Yun Liu, Sifei Yu, Zitao Li, Jiangjun Ma, Yannan Zhang, Hui Wang, Binyan Yang, Changyou Wu

**Affiliations:** Institute of Immunology, Zhongshan School of Medicine; Key Laboratory of Tropical Disease Control Research of Ministry of Education, Sun Yat-sen University, Guangzhou, China; University of Southern California, United States of America

## Abstract

IL-21 has pleiotropic effects on innate and adaptive immune response, and plays an important role in the development of autoimmune disease and antitumor activity. It has been reported that IL-21 is produced by CD4^+^ T cells and NKT cells. However, the differentiation of IL-21-producing CD4^+^ T cells in humans remains largely unclear. In the present study, we showed that cytokines of IL-1β, IL-6 or IL-21 induced differentiation of human IL-21-producing CD4^+^ T cells, and TGF-β enhanced the effect of inflammatory cytokines on the development of IL-21-producing CD4^+^ T cells. Furthermore, we found that the majority of IL-21-producing cells were distinct from Th17 cells and Th1 cells since they did not co-express IL-17 and IFN-γ. TGF-β significantly inhibited the production of IFN-γ and enhanced the effect of IL-21 on the development of IL-21-producing CD4^+^ T cells. In addition, we found that IL-21 inhibited the differentiation of CD4^+^ Foxp3^+^ T cells induced by TGF-β. Further study indicated that IL-21 induced phosphorylation of transcriptional factors of STAT1, STAT3 and STAT5, and TGF-β induced phosphorylation of Smad3 in CD4^+^ T cells. Taken together, our data indicated that TGF-β enhanced IL-21-induced differentiation of IL-21-producing CD4^+^ T cells, and the majority of IL-21-producing cells were different from Th17 and Th1 cells. Our results provide a new sight regarding the differentiation of human CD4^+^ T cells.

## Introduction

Specific immune response of T cells is the core strength to fight against invading pathogens in the immune system. On activation, naïve T cells differentiate into effector T-cell subsets with specific cytokine production and specialized effector functions. A subset of T cells distinct from T helper (Th) 1 and Th2 cells producing interleukin-17 (IL-17) was defined as Th17 cells [1], [2]. Th17 cells mediate neutrophil differentiation and infiltration during various infections [3].

Interleukin-21 (IL-21) exerts critical functions in T helper type 17 (Th17) cell developments [4], [5]. IL-21 is a four-helix-bundle type I cytokine with significant homology to IL-2, IL-15 and IL-7 [6]. IL-21 has been demonstrated to be expressed by various of T helper cell subsets, including T follicular helper cells, T helper type 1(Th1), Th2, Th17 cells and natural killer T (NKT) cells [4], [5], [7]. IL-21 exhibits pleiotropic effects on the proliferation, differentiation and effective function of T, B, NK and dendritic cells [8]–[15]. The regulatory activity of IL-21 is modulated by the differentiation state of its target cells as well as by other cytokines or co-stimulatory molecules.

TGF-β is a multi-functional pleiotropic cytokine, displaying a significant role in embryogenesis development, tissue renewal and regulation of the immune system. TGF-β is known to regulate many branches of hematopoiesis, affect differentiation and proliferation of hematopoietic stem cells as well as progenitor cells of erythrocytes, macrophages, dendritic cells and other lineages [16]–[18]. TGF-β also influences T cells at several different phases of development/differentiation, including controlling formation of both inflammatory Th17 cells and Foxp3^+^ regulatory T cells [19]–[24]. Furthermore, TGF-β reprograms the differentiation of T helper 2 cells and promotes an interleukin 9-producing subset [25].

It has been demonstrated that in mice IL-21-producing CD4^+^ T cells exhibit distinct characteristics from Th17 cells and develop preferentially in an IL-6-rich environment devoid of TGF-β [26]. However, the characterization of human IL-21-producing T cells and the contribution of IL-21 and TGF-β in the differentiation of IL-21-producing T cells remain largely unexamined and elusive.

With this information, we carried out studies on the function of cytokines in the development of IL-21-producing CD4^+^ T cells. We found that IL-21 enhanced the production of IL-21 by human naïve CD4^+^ T cells and TGF-β strengthened the effect of IL-21 on the development of IL-21-producing CD4^+^ cells with different signal pathways.

## Materials and Methods

### Subjects

Umbilical cord blood from healthy full-term newborn infants was collected from the Secondary Affiliated Hospital of Sun Yat-sen University**,** China. The parents/guardian of the newborns gave written consent and the study was approved by the Medical School Review Board at Zhongshan School of Medicine, Sun Yat-sen University, China.

### Monoclonal Abs

The following antibodies were used for cell surface and intracellular staining: FITC-labeled anti-CD4, PerCP-labeled anti-CD4, APC-labeled anti-interferon-γ (IFN-γ), FITC-labeled anti-IFN-γ, APC-labeled anti-Foxp3, PE-labeled anti-phosphor-STAT1, PE-labeled anti-phosphor-STAT3, FITC-labeled anti-phosphor-STAT4, FITC-labeled anti-phosphor-STAT5, APC-labeled anti- phosphor-STAT6, isotype-matched control antibodies were purchased from BD Bioscience PharMingen (San Jose, CA, USA). APC-labeled anti-IL-21 and PE-labeled anti-IL-21 were purchased from R&D Systems (Minneapolis, MN). PE-labeled anti-IL-17, APC-labeled anti-Foxp3 mAbs and Foxp3 staining buffers were obtained from eBioscience (San Diego, CA, USA). Purified anti-CD3, anti-CD28 and anti-IFN-γ monoclonal antibodies (mAbs) were purchased from BD Bioscience PharMingen (San Jose, CA, USA). The following antibodies were used for western blot: rabbit anti-total-Stats, phospho-Stats, total-Smad3, phospho-Smad3 and mouse anti-rabbit HRP were purchased from Cell Signaling Technology (Cambridge, MA). c-Maf (M-153) was purchased from Santa Cruz Biotechnology (Santa Cruz, CA).

### Preparation of CBMCs

For preparation of cord blood mononuclear cells (CBMCs), heparinized cord blood was mixed sufficiently with Dextran 500 solution (GE Healthcare Bio-Sciences, Uppsala, Sweden) and incubated at 37°C in a 5% CO_2_ incubator for 30 min to remove erythrocytes. CBMCs were obtained by Ficoll-Hypaque density gradient centrifugation. Cells were suspended at a concentration of 2×10^6^/mL in complete RPMI 1640 medium (Gibco, Grand Island, NY, USA) supplemented with 10% FCS (Sijiqing, China), 100 U/mL penicillin, 100 µg/mL streptomycin, 50 µM 2-mercaptoethanol and 2 mM L-glutamine (all from Gibco BRL).

### Isolation of T cell Subsets

Naïve CD4^+^ T cells were isolated from CBMCs by positive selection with anti-CD4 microbeads (Miltenyi Biotec, Bergisch Gladbach, Germany). The purity of cells assessed by flow cytometry (FACS Calibur, Becton Dickinson, San Jose, CA) exceeded 98% for each T subset. Cells were suspended at a concentration of 0.5×10^6^/mL in complete RPMI 1640 medium.

### Cell Culture Conditions

Naïve CD4^+^ T cells were stimulated with immobilized anti-CD3 (1 µg/mL) plus anti-CD28 (1 µg/mL) mAbs. IL-1β (10 ng/mL; Peprotech, Rocky Hill, NJ, USA), IL-6 (30 ng/mL; Peprotech), IL-21 (50 ng/mL; Peprotech), TGF-β (2 ng/mL; Peprotech), IL-21 plus TGF-β or anti-IFN-γ (10 µg/mL) were added to the cultures. The cells were harvested and re-stimulated with PMA (20 ng/mL; Sigma-Aldrich) plus ionomycin (1 µg/mL; Sigma-Aldrich) in the presence or absence of Brefeldin A (10 µg/mL; P310, Sigam-Aldrich) and used for flow cytometry analysis or RNA extraction. Culture supernatants for 48 h were used for cytokines measurement by ELISA.

### Detection of Phosphorylated STATs by Flow Cytometry

Naïve CD4^+^ T cells were activated with immobilized anti-CD3 and anti-CD28 mAbs for 2 d, rested overnight and re-stimulated with or without IL-21 (50 ng/mL), TGF-β (2 ng/mL) or IL-21 plus TGF-β for 15 min. The cells were fixed in 2% formaldehyde, permeabilized in 90% methanol and labeled with anti-phosphor-STAT1, -STAT3, -STAT4, -STAT5 or -STAT6 mAbs.

### Cell Surface and Intracellular Cytokine Staining

Purified naïve CD4^+^ T cells from CBMCs were stimulated as described above and re-stimulated with PMA plus ionomycin for 5 h in the presence of Brefeldin A. Cells were washed, fixed, permeabilized and stained with detected mAbs for 30 min at 4°C. Isotype-matched control antibodies were added to the cells as well. After intracellular staining, cells were washed and suspended in PBS. Foxp3 staining was conducted according to the Foxp3 staining protocol recommended by eBioscience. Flow cytometry was performed using a BD FACS Calibur cytometer. Lymphocytes were gated on forward and side scatter profiles and analyzed using FlowJo software (Treestar, San Carlos, CA).

### PCR for IL-21

Purified naïve CD4^+^ T cells from CBMCs were stimulated and rested as described above and re-stimulated with PMA plus ionomycin for 5 h. Total RNA was extracted by TRIzol (Invitrogen, Carlsbad, CA, USA) according to the manufacturer's instructions. Reverse transcription of total RNA was performed at 37°C using the ReactionReady™ First Strand cDNA Synthesis Kit (Invitrogen). Amplification of cDNA was conducted in a DNA thermal cycler (Biometra, Germany) at the following conditions: denaturation 45 s at 94°C, annealing 45 s at 65°C for GAPDH and IL-21, followed by 1 min of elongation at 72°C. PCR rounds were repeated for 25 to 35 cycles each for both GAPDH and IL-21. The following sense and antisense primers for each molecule were used: IL-21 forward: 5′-CGT CTG CCC TAT CAA CTT TCG-3′; reverse: 5′-GAG AAA CGG CTA CCA CAT CCA-3′. Glyceraldehyde-3-phosphate dehydrogenase (GAPDH) forward: 5′-GCA TGG CCT TCC GTG TCC-3′; reverse: 5′-TGA GTG TGG CAG GGA CTC-3′. The ratio of IL-21 over GAPDH was calculated according to the relative intensities of the bands revealed under UV illumination with Bio-1D software (Vilber Lourmat, Marne la Vallee, France).

### Western Blotting for Total or Phosphorylated-STATs, Smad3

Purified naïve CD4^+^ T cells from CBMCs were activated with immobilized anti-CD3 and anti-CD28 mAbs for 2 d, rested overnight and re-stimulated with or without IL-21 (50 ng/mL), TGF-β (2 ng/mL; Peprotech) or IL-21 plus TGF-β for 15 min at 37°C. The cells were lysed in a lysis buffer and cell lysates were separated on a 10% SDS-PAGE and transferred to polyvinylidene difluoride membrane (Millipore). The membrane was blocked, probed with primary specific antibodies and with appropriate secondary antibodies conjugated to HRP and visualized with the ECL detection system according to the manufacturer’s instructions.

### ELISA

Cell-free culture supernatants were harvested and assayed by ELISA for the production of IL-21 (Peprotech), IL-17 (eBioscience) and IFN-γ (BD Bioscience PharMingen) according to the manufacturer’s protocols, respectively.

### Statistical Analysis

Data are presented as the mean ± SD values. Comparison between two groups was performed by unpaired or paired Student’s *t*-tests. A value of *P*<0.05 was considered significant.

## Results

### Cytokines Induced the Expression of Interleukin-21 (IL-21) by Human Naïve CD4^+^ T cells

**Figure 1:**
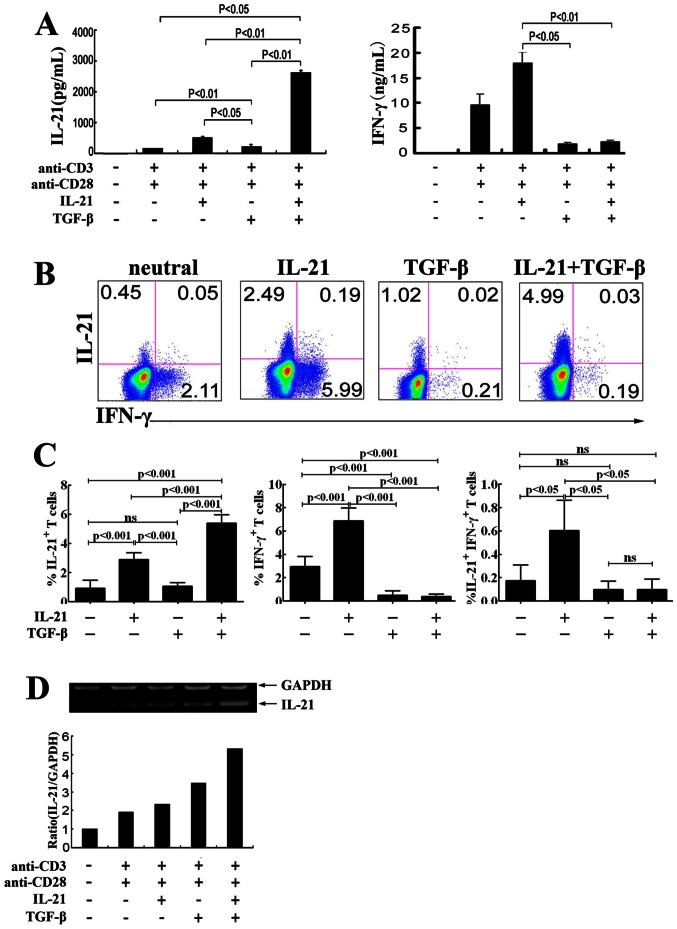
TGF-β enhances the effect of IL-21 on the development of IL-21-producing CD4^+^ T cells. Naïve CD4^+^ T cells from CBMCs were stimulated for 3 days with immobilized anti-CD3 and anti-CD28 mAbs (neutral condition) in the presence or absence of IL-21, TGF-β or IL-21 plus TGF-β. The cells were re-stimulated with PMA and ionomycin. A. The levels of IL-21 and IFN-γ were quantified by ELISA. Statistical results were mean±SD from four independent experiments. P<0.05 and P<0.01 were considered significant. B. IL-21 and IFN-γ-producing cells were determined by flow cytometry. C. Statistical data were mean±SD from four independent experiments as described in B. P<0.05 and P<0.01 were considered significant. D. The levels of IL-21 and GAPDH mRNA were determined by PCR. Data were representative of three separate experiments with similar results.

To evaluate the production of IL-21 under different stimulation condition, purified naïve CD4^+^ T cells from CBMCs were stimulated with immobilized anti-CD3 and anti-CD28 in the absence or presence of different cytokines. The results showed that IL-1β, IL-6 or IL-1β plus IL-6 induced the production of IL-21 (data not shown). Interestingly, TGF-β enhanced IL-1β and IL-6 to induce the production of IL-21. Simultaneously, we found that IL-21 enhanced the production of IFN-γ and TGF-β that significantly inhibited the production of IFN-γ (Fig. 1A, 1B and 1C). In addition, approximately 20% CD4^+^IL-21^+^ T cells and 1% CD4^+^IL-21^+^ T cells co-expressed IFN-γ under the stimulation with IL-6 and with TGF-β, IL-1β plus IL-6 respectively (data not shown). Taken together, these results indicated that naïve CD4^+^ T cells could be induced to produce IL-21 under the stimulation of different cytokines.

It has been reported that IL-21 induced the development of IL-21-producing CD4^+^ T cells in mice [26]. To investigate whether IL-21 has any effect on the development of IL-21-producing T cells in humans, we stimulated naïve CD4^+^ T cells from CBMCs with immobizied anti-CD3 plus anti-CD28 in the presence or absence of IL-21 plus TGF-β (primary stimulation), the cells were rested and re-stimulated with PMA plus ionomycin (secondary stimulation) for the production of cytokines. The results showed that anti-CD3 plus anti-CD28 induced a low level of IL-21 production (Fig. 1A). Addition of IL-21 or TGF-β into cultures resulted in elevated levels of IL-21 production. Notably, IL-21 plus TGF-β induced the highest production of IL-21 by naïve CD4^+^ T cells (Fig. 1A). In addition, IL-21 plus TGF-β enhanced the production of IL-21 but inhibited the induction of IFN-γ in protein level (Fig. 1A). The similar results were observed by FACS (Fig. 1B and 1C) and by PCR (Fig. 1D). Taken together, these results suggested that IL-21 had a synergistic effect with TGF-β in the production of IL-21 by naïve CD4^+^ T cells from CBMCs, and the subset of IL-21-producing cells was distinct from Th1 and Th17 subpopulations.

### IL-21 Plus TGF-β Induced the Production of IL-21 in a does- and Time- Dependent Manner

**Figure 2:**
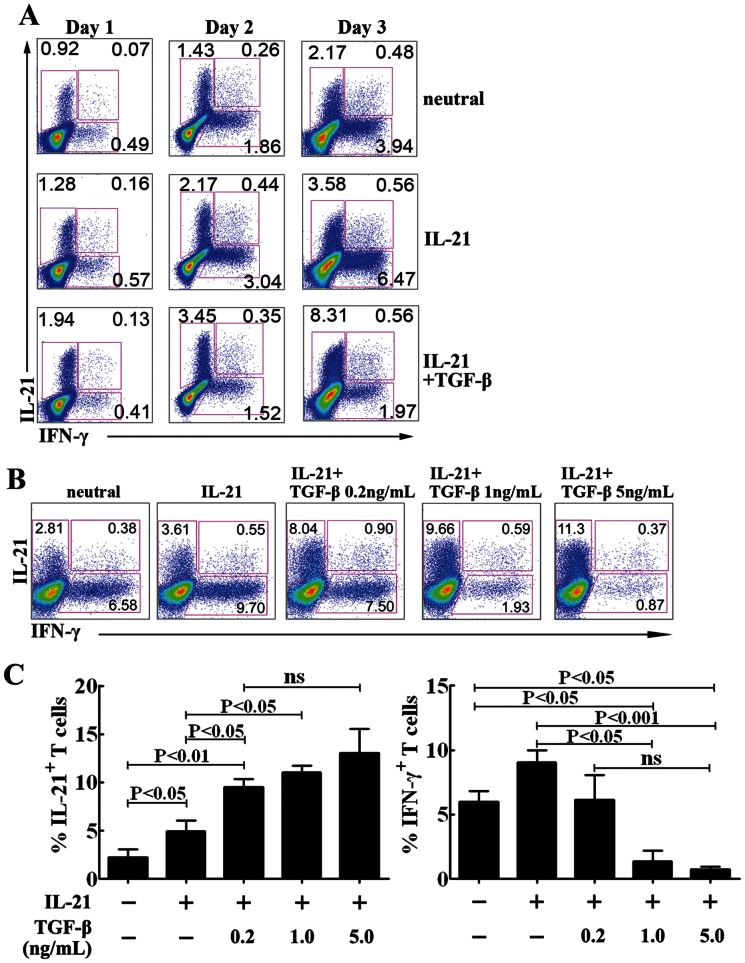
The kinetics of IL-21 and IFN-γ production by CD4^+^ T cells. A. Naïve CD4^+^ T cells from CBMCs were stimulated with immobilized anti-CD3 and anti-CD28 mAbs (neutral condition) in the presence or absence of IL-21 or IL-21 plus TGF-β. The cells were re-stimulated with PMA plus ionomycin after the primary culture and harvested at different times. The expression of IL-21 and IFN-γ was determined by flow cytometry. Data were representative of four separate experiments with similar results. B. Naïve CD4^+^ T cells from CBMCs were stimulated for 3 days with immobilized anti-CD3 and anti-CD28 mAbs (neutral condition) in the presence or absence of IL-21 or IL-21 plus TGF-β at the indicated concentrations. The cells were re-stimulated with PMA plus ionomycin. IL-21 and IFN-γ-producing cells were determined by flow cytometry. C. Statistical data shown were mean±SD from three separate experiments as described in B. P<0.05 and P<0.01 were considered significant.

To further determine the effect of IL-21 plus TGF-β in the production of IL-21, purified naïve CD4^+^ T cells from CBMCs were stimulated with anti-CD3 plus anti-CD28 in presence or absence of IL-21 or IL-21 plus TGF-β. The cells were collected at different time points and the production of IL-21 and IFN-γ were detected by flow cytometry. As shown in Fig. 2, a low frequency of IL-21 was induced following the stimulation with IL-21 plus TGF-β at day 1 and markedly increased at day 3 (Fig. 2A). Purified naïve CD4^+^ T cells from CBMCs were cultured with or without IL-21 plus TGF-β at the different concentrations in the presence of anti-CD3 and anti-CD28. The expression of IL-21 and IFN-γ were determined by flow cytometry. The results showed that IL-21 plus TGF-β could induce IL-21 production from CD4^+^ T cells in a dose-dependent manner (Fig. 2B and 2C).

### Neutralizing of IFN-γ Enhanced the Production of IL-21 by Naïve CD4^+^ T cells

**Figure 3:**
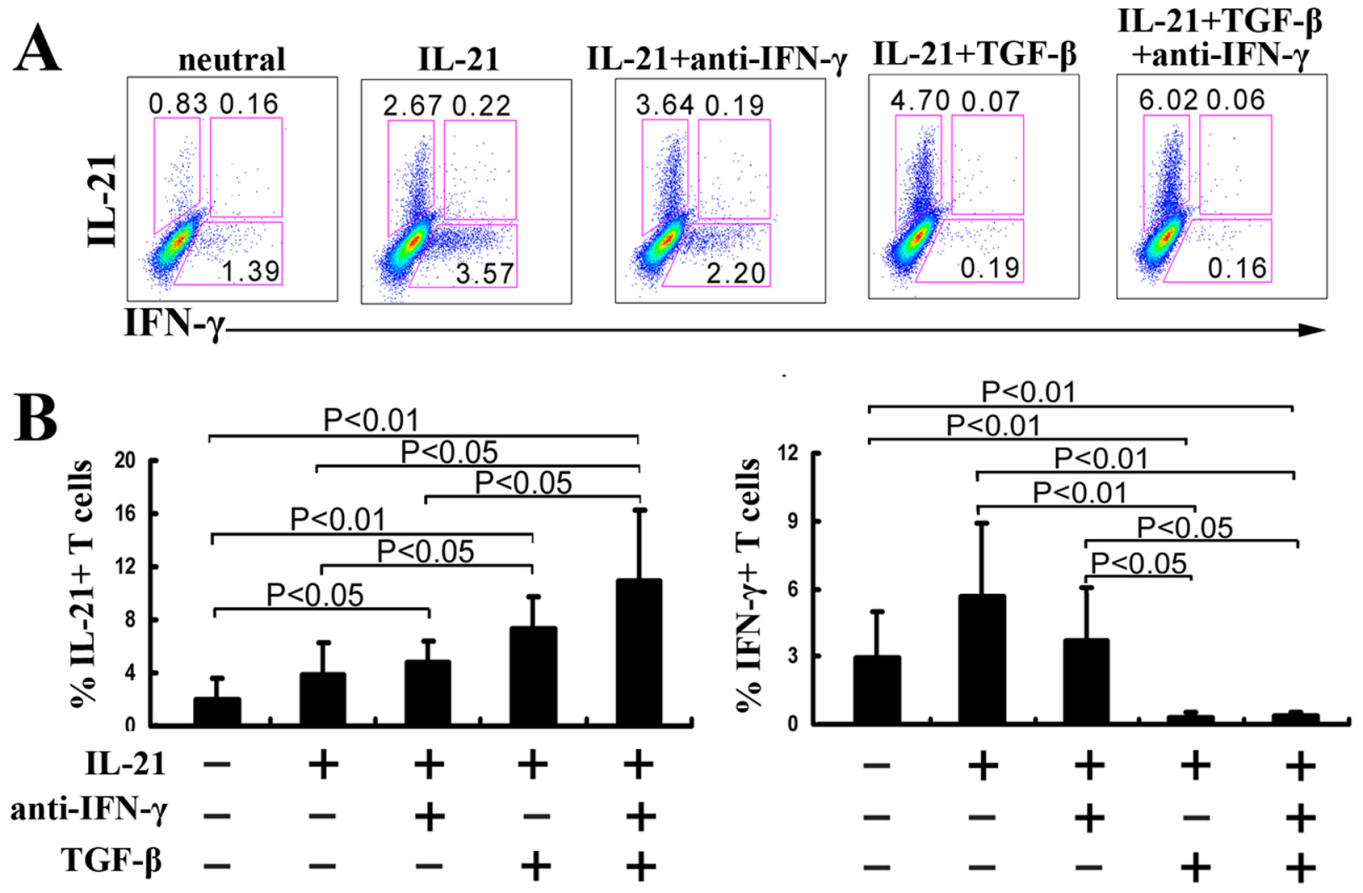
Neutralizing of IFN-γ enhances the production of IL-21 from CD4^+^ T cells. A. Naïve CD4^+^ T cells from CBMCs were stimulated for 3 d with immobilized anti-CD3 and anti-CD28 mAbs (neutral condition) in the presence or absence of IL-21, IL-21 plus TGF-β and the mAb against IFN-γ. The cells were re-stimulated with PMA and ionomycin. IL-21 and IFN-γ-producing cells were determined by flow cytometry. Data were representative of four independent experiments with similar results. B. Statistical results shown were mean±SD from four independent experiments as described in A. P<0.05 was considered significant.

TGF-β has been shown to inhibit IFN-γ production [16], [17]. We examined the effect of anti-IFN-γ antibody on the differentiation of IL-21-producing CD4^+^ T cells in the presence of IL-21. The results showed that a neutralizing antibody against IFN-γ increased the number of IL-21-producing CD4^+^ T cells with the decreased number of IFN-γ-producing CD4^+^ T cells. Moreover, anti-IFN-γ could further increase the number of IL-21-producing CD4^+^ T cells in the presence of IL-21 plus TGF-β (Fig. 3A and 3B). These results suggested that TGF-β promoted the differentiation of IL-21-producing CD4^+^ T cells in part via its suppressive effect on IFN-γ production.

### TGF-β-enhanced the Expression of CD4^+^Foxp3^+^ T cells was Inhibited by IL-21

**Figure 4:**
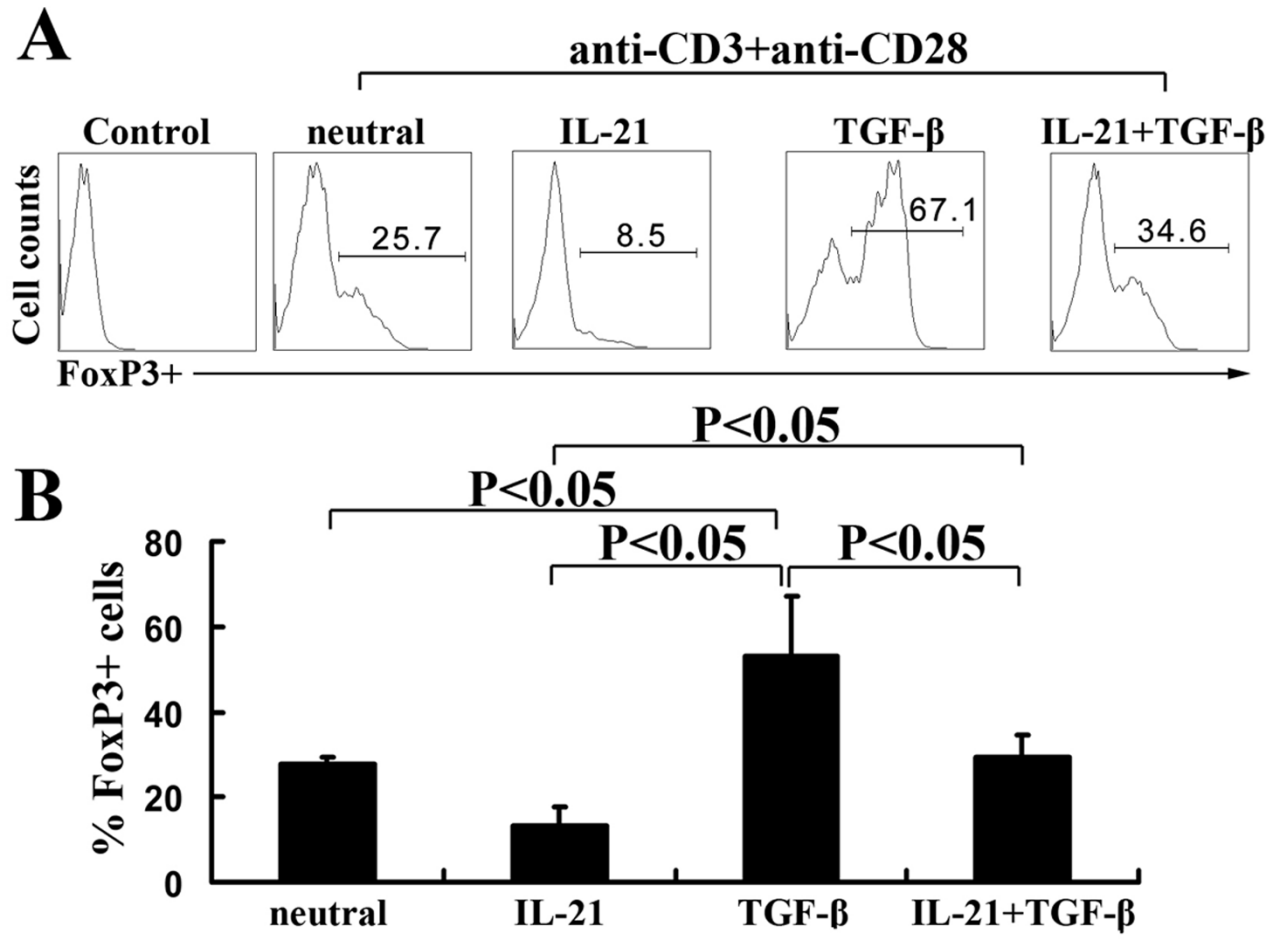
IL-21 significantly inhibits the expression of Foxp3^+^ T cells by TGF-β-induced naïve CD4^+^ T cells. A. Naïve CD4^+^ T cells from CBMCs were stimulated for 3 d with immobilized anti-CD3 and anti-CD28 mAbs (neutral condition) in the presence or absence of IL-21, TGF-β or IL-21 plus TGF-β. The cells were re-stimulated with PMA plus ionomycin. The expression of Foxp3 was determined by flow cytometry. Data were representative of three independent experiments with similar results. B. Statistical results shown were mean±SD from three independent experiments as described in A. P<0.05 was considered significant.

Since TGF-β induced the expression of Foxp3, we next assessed the Foxp3 expression under the different culture conditions. Naïve CD4^+^ T cells were activated with the stimulation of anti-CD3 and anti-CD28 in the presence or absence of TGF-β, IL-21 or TGF-β plus IL-21. The results showed that stimulation of naïve CD4^+^ T cells with anti-CD3 and anti-CD28 induced the expression of Foxp3. Addition of IL-21 decreased the frequency of Foxp3^+^ T cells. As expected, TGF-β greatly enhanced the expression of Foxp3. Addition of IL-21 into cultures inhibited the expression of Foxp3 to 50 percent (Fig. 4A and 4B). Collectively, these results indicated that IL-21 significantly decreased the frequency of CD4^+^Foxp3^+^ T cells induced by TGF-β, but the mechanism and effect remained unclear.

### IL-21 and TGF-β Induced the Phosphorylation of STATs and Smad3

**Figure 5:**
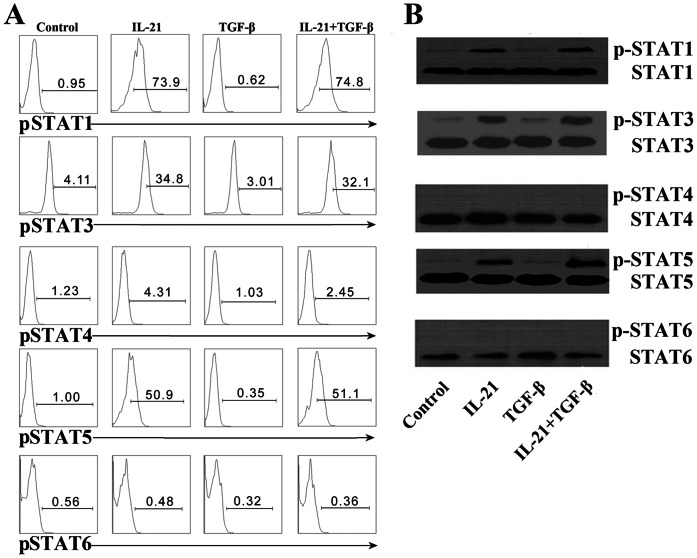
Signals of STAT1, STAT3 and STAT5 but not STAT4 and STAT6 mediate the induction of IL-21 by CD4^+^ T cells. Naïve CD4^+^ T cells from CBMCs were stimulated for 2 d with immobilized anti-CD3 and anti-CD28 mAbs (neutral condition), rested overnight and re-stimulated for 15 min with or without IL-21, TGF-β or IL-21 plus TGF-β. A. The cells were harvested and the expression of phosphorylated STAT1, STAT3, STAT4, STAT5 or STAT6 was determined by flow cytometry. The histograms represented the expression of phosphorylated STATs from four separate experiments with similar results. B. The cells were lysed and subjected to western blotting. A single membrane and phosphotyrosine specific antibodies had been used. The total STATs were as the positive controls. Data were representative of three independent experiments with similar results.

**Figure 6:**
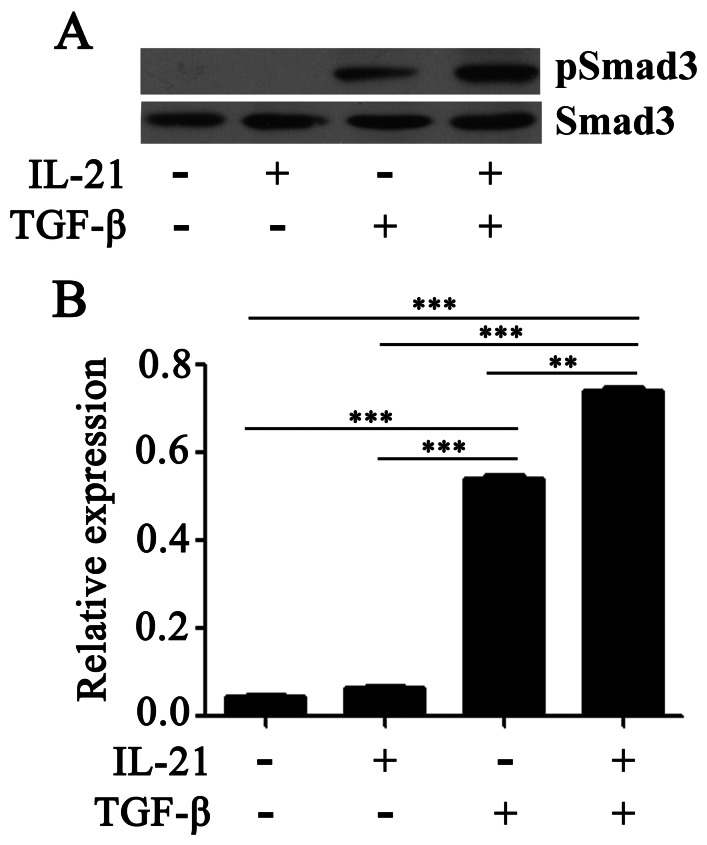
Smad3 is involved in TGF-β-mediated the up-regulation of IL-21-producing cells. Naïve CD4^+^ T cells from CBMCs were stimulated for 2 d with immobilized anti-CD3 and anti-CD28 mAbs (neutral condition), rested overnight and re-stimulated for 15 min with or without IL-21, TGF-β or IL-21 plus TGF-β. A. The cells were lysed and subjected to western blotting. A single membrane and phosphotyrosine specific antibodies had been used. The total Smad3 were as the positive controls. Data were representative of three separate experiments with similar results. B. The ratio of pSmad3 to tSmad3 was quantified by desitometry, and statistical data shown were mean±SD from three independent experiments as described in A. P<0.05 was considered significant.

To identify the signaling pathways, we cultured naïve CD4^+^ T cells in the presence of IL-21, TGF-β, and IL-21 plus TGF-β. The phosphorylation of STATs was analyzed by flow cytometry (Fig. 5A) and western blotting (Fig. 5B). Consistent with our previous results, stimulation of CD4^+^ T cells with IL-21 resulted in phosphorylation of STAT1, 3 and 5. TGF-β did not induce the phosphorylation of STATs (Fig. 5A and 5B). We then assessed the activation of Smad3 by western blotting. As shown in Fig. 6, IL-21 alone did not activate Smad3, but TGF-β increased phosphorylation of Smad3. Addition of IL-21 into culture increased TGF-β-induced phosphorylation of Smad3 but slightly decreased expression of c-Maf (data not shown).

## Discussion

IL-21 is a pleiotropic cytokine that has a broad range of activities on both innate and adaptive immune cells. IL-21 is produced by NKT and CD4^+^ T cells and plays an important role in the development of autoimmune diseases and is confirmed as a potent antitumor agent in animal models [27]–[33]. However, the differentiation of IL-21-producing cells in humans remains largely unclear. It is well known that TGF-β exerts critical functions in Th17 cell development in mouse models [19]–[22]. The effect of TGF-β on the differentiation of IL-21-producing CD4^+^ T cells in humans is not well understood.

In this study, we found that IL-1β or IL-6 alone could potently induce the development of IL-21-producing CD4^+^ T cells in humans following the stimulation with anti-CD3 and anti-CD28 mAbs (data not shown). It has been reported that in mouse model, TGF-β inhibited the development of IL-21-producing CD4^+^ T cells [26]. In contrast with the results, we demonstrated that TGF-β enhanced the effect of inflammatory cytokines on the development of IL-21-producing CD4^+^ T cells. Furthermore, our data indicated that IL-21 itself induced the development of IL-21-producing CD4^+^ T cells, and the effect of IL-21 was enhanced by TGF-β in a dose- and time- dependent manner. The results suggested that the mechanisms underlying the differentiation of IL-21-producing CD4^+^ T cells in humans and mouse were different. It has been demonstrated in mouse model, c-Maf directly binds to and activates IL-21P and the CNS-2 enhancer through MARE sites and induces IL-21 production. TGF-β suppresses c-Maf-induced IL-21 production in CD4^+^ T cells [34]. Previous studies showed that IL-21 and TGF-β exerted critical functions in the development of Th17 cells [4], [5]. Our results demonstrated IL-21 together with TGF-β induced the development of Th17 cells (data not shown) and IL-21-producing CD4^+^ T cells. However, the majority of IL-21-producing cells did not co-express IL-17 and IFN-γ, indicating that the IL-21-producing CD4^+^ T cells were distinct from Th17 cells and Th1 cells. On the other hand, it has been demonstrated that IL-21 suppresses Foxp3 expression [35]. Consistent with the results, we found that IL-21 decreased the frequency of CD4^+^ Foxp3^+^ T cells. TGF-β induced Foxp3 expression and generated Treg cells. These findings suggest that IL-21 and TGF-β regulate the balance of IL-21-producing CD4^+^ T cells, Th17 cells and Treg cells in an environment.

TGF-β has been shown to inhibit IFN-γ production [16], [17]. We demonstrated that neutralizing of IFN-γ enhanced the effect of IL-21 on the development of IL-21-producing CD4^+^ T cells. TGF-β might promote the differentiation of IL-21-producing CD4^+^ T cells in part via its suppressive effect on IFN-γ production.

We next examined the mechanisms underlying the transcriptional regulation of IL-21 production in CD4^+^ T cells. Depending on the cell type, IL-21 activates Janus family tyrosine kinases JAK1 and JAK3 to the activation of different STATs signals [8], [9]. Our data demonstrated that treatment of activated CD4^+^ T cells with IL-21 increased phosphorylation of STAT1, STAT3 and STAT5, thus suggesting that IL-21 might induce IL-21 production by activating target genes downstream of STAT1, STAT3 and STAT5 in the activated naïve CD4^+^ T cells. Collectively, IL-21 inhibited the expression of Foxp3 and generation of Treg cells which might be different from the signaling pathway of STATs. TGF-β did not influence the levels of phosphorylation of STATs, but induced high phosphorylation of Smad3 in activated naïve CD4^+^ T cells. The addition of IL-21 enhanced the level of phosphorylation of Smad3. c-Maf is another critical transcription factor that activates the promoter and enhancer of IL-21 gene, our results showed that co-stimulation with IL-21 and TGF-β slightly decreased the expression of c-Maf compaired with stimulation with TGF-β alone in CD4^+^ T cells (data not shown). These results indicated that TGF-β-Smad3 signaling might involve in the development of IL-21-producing CD4^+^ T cells.

In summary, our data demonstrated that as an autocrine growth factor, IL-21 induced the differentiation of IL-21-producing CD4^+^ T cells. In contrast with the results in mouse model, we found that TGF-β enhanced the IL-21-induced development of IL-21-producing CD4^+^ T cells in humans. The IL-21-producing CD4^+^ T cells were distinct from Th17 cells and Th1 cells. IL-21 and TGF-β could regulate the balance of IL-21-producing CD4^+^ T cells, Th17 cells and Treg cells in an environment. Our data added a new insight into the differentiation of IL-21-producing CD4^+^ T cells in humans and enhanced our understanding of the regulation of this new population of CD4^+^ T cells.
